# Facile Construction of Chestnut‐Like Structural Fireproof PDMS/Mxene@BN for Advanced Thermal Management and Electromagnetic Shielding Applications

**DOI:** 10.1002/advs.202307482

**Published:** 2024-02-11

**Authors:** Fuping Bian, Rui Huang, Xiaobin Li, Jiwen Hu, Shudong Lin

**Affiliations:** ^1^ Guangzhou Institute of Chemistry Chinese Academy of Sciences Guangzhou 510650 P. R. China; ^2^ University of Chinese Academy of Sciences Beijing 100049 P. R. China; ^3^ CAS Engineering Laboratory for Special Fine Chemicals Guangzhou 510650 P. R. China; ^4^ CASH GCC Shaoguan Research Institute of Advanced Materials Nanxiong 512400 P. R. China; ^5^ CASH GCC Fine Chemicals Incubator (Nanxiong) Co., Ltd Nanxiong 512400 P. R. China

**Keywords:** boron nitride, electromagnetic shielding performance, flame retardancy, MXene, thermal conductivity

## Abstract

Composite polymer materials featured superior thermal conductivity, flame retardancy, and electromagnetic shielding performance are increasingly in demand due to the rapid development of highly miniaturized, portable, and flexible electronic devices. Herein, a facile and green ball milling shear method is utilized for generating MXene@Boron nitride (MXene@BN). The multi‐functional fillers (MXene@BN) are constructed and incorporated into polydimethylsiloxane (PDMS) to prepare a multifunctional composite (PDMS/MXene@BN) for achieving improved electromagnetic interference (EMI) shielding performance and thermal conductivity as well as flame retardancy simultaneously. When the PDMS/MXene@BN composite has a MXene@BN loading of 2.4 wt.%, it exhibits a high thermal conductivity of 0.59 W m^−1^K^−1^, which is 210% higher than that of the pure PDMS matrix. This is attributed to its unique chestnut‐like double‐layer structure. With a smoke production rate (SPR) of 0.04 m^2^ s^−1^ and total smoke production (TSP) of 3.51 m^2^, PDMS/MXene@BN 2.4 composite exhibits superb smoke suppression properties. These SPR and TSP values are 63.20% and 63.50% lower than the corresponding values of pure PDMS. Moreover, the EMI SE of the PDMS/MXene@BN 2.4 can reach 26.3 dB at 8.5 GHz. The work reported herein provides valuable insight into developing composites with multiple functions, which show strong potential for application in advanced packaging materials.

## Graphical abstract

A facile and green ball milling shear method is utilized for generating MXene@BN. The multi‐functional fillers (MXene@BN) are constructed and incorporated into polydimethylsiloxane (PDMS) to prepare a multifunctional‐composites (PDMS/MXene@BN) for achieving improved electromagnetic interference (EMI) shielding performance and enhanced thermal conductivity as well as flame retardancy simultaneously. This work provides a biomimetic method for manufacturing high‐performance composite materials with excellent flame retardancy and thermal conductivity as well as EMI shielding and has great prospects for applications in electronic devices.

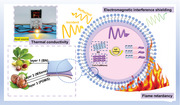

## 1 Introduction

Electronic equipment is increasingly miniaturized and highly integrated, and this trend has accelerated due to the deployment and ongoing development of 5G communication technology. Thus, electronic components with improved comprehensive performance are needed.^[^ ^1^ ^]^ In particular, there is a more urgent need for polymeric composites that have properties such as heat dissipation, flame retardancy, as well as electromagnetic interference (EMI) shielding properties applied to polymeric composites for electronic devices.^[^ ^2^ ^]^ Heat is produced during the operation of electronic components which can degrade the performance of nearby devices and can pose serious fire hazards.^[^ ^3^ ^]^ Meanwhile, undesirable electromagnetic waves can affect the performance of electronic components during operation and generate noise pollution. To design electronic devices with efficient thermal management, good flame retardancy, and electromagnetic compatibility (EMC), materials that combine a number of features, including high thermal conductivity and flame retardancy as well as good EMI shielding, are urgently required.^[^ ^4^ ^]^ Various polymers with improved thermal conductivity have recently been developed by using a variety of highly thermally conductive fillers. These fillers include Boron nitride (BN),^[^ ^5^, ^6^ ^]^ graphene,^[^ ^7^, ^8^ ^]^ Ti_3_C_2_T_X_ (MXene),^[^ ^9^, ^10^ ^]^ carbon fiber,^[^ ^11^, ^12^ ^]^ and silicon carbide nanowires.^[^ ^13^ ^]^

Among them, MXene are a new family represented by 2D materials. General formula M_n+1_X_n_T_x_ (*n* = 1–4), where T is surface end groups (–O–, –OH and/or –F), X represents carbon and/or nitrogen, M represents early transition metal group.^[^ ^14^ ^]^ A considerable number of surface functional groups enable MXene to be functionalized with organic compounds or employed for the preparation of nanocomposites.^[^ ^15^ ^]^ Up to now, MXene has attracted a great deal of attention in the scientific community for its unique layered structure and excellent chemical, physical and thermal properties. It was synthesized by etching Ti_3_AlC_2_ (MAX) powder in HCl and LiF solutions. Single layer/few layers of MXene were further obtained by ultrasonic treatment.^[^ ^16^ ^]^ Shahzad et al. firstly reported the potential of several MXene and their polymer composites for EMI shielding. Moreover, Zha et al. demonstrate that the MXene have good thermal conductivity are higher than those of most metals and semiconducting low‐dimensional materials by first‐principles calculations.^[^ ^17^ ^]^ However, MXene also has shortcomings such as low insulation and poor air stability. This makes it unable to meet the actual application needs of electronic components.

In contrast,^[^ ^18^ ^]^ BN has the advantages of good insulating properties, a low expansion coefficient, etc.^[^ ^19^ ^]^ Zhang and co‐workers fabricated highly thermally conductive and electrically insulating PDMS composites via the combination of BN and CNC. However, they found that BN overloading can lead to a decrease in the mechanical properties of composites.^[^ ^20^ ^]^ The higher interfacial thermal resistance is due to poorer interaction between BN and polymer. Although surface functionalization of BN has been used to improve these interactions, functionalization of BN serves to disrupt its crystal structure.^[^ ^21^ ^]^ This will have a serious impact on its overall performance. Based on the respective advantages and disadvantages of MXene and BN, it will be an important breakthrough if the two can be combined to complement each other and prepare high‐performance functional composite fillers with insulation, thermal conductivity, and high electromagnetic shielding properties.

In practical electrical applications, it is often necessary to add functional fillers to polymer resins to prepare high‐performance composite materials. Polydimethylsiloxane (PDMS) is commonly used in the field of electrical packaging and has many desirable properties, such as providing electrical insulation properties, high and low temperature resistance, biocompatibility, chemical inertness and other properties.^[^ ^22^ ^]^ Wang et al. prepared a coating with superhydrophobic and flame‐retardant properties by covering STA‐ATH particles on the surface of lignocellulose composites coated with PDMS.^[^ ^23^ ^]^ The PDMS composite synthesized by Song et al. possess an orderedi‐scale MXene and PEI‐Cu^2+^ are conducive to excellent flame retardancy structure.^[^ ^24^ ^]^ However, a key shortcoming of PDMS is that it has poor thermal conductivity.^[^ ^25^, ^26^, ^27^ ^]^ Multiple studies have confirmed that the use of multiple fillers in a thermally conductive composite polymer can lead to a synergistic effect which can provide a greater improvement of the thermal conductivity than can be achieved with a single filler. Yang et al. blended single‐walled nanotubes and graphene into epoxy resin. Through the 3D thermal conductivity network constructed by the nanotubes and graphene, the thermal conductivity of the composite material was increased by 146.9%.^[^ ^28^ ^]^ Liu et al. prepared a highly thermally conductive and insulating flexible composite film by introducing SCF and BN particles into a PDMS matrix via spin coating.^[^ ^29^ ^]^ Jiao et al. used a simple vacuum‐assisted filtration method to prepare a flexible cellulose nanofiber (CNF)/nanodiamond (ND)/MXene (CNM) composite film that exhibits a high thermal conductivity of 17.43 W m^−1^K^−1^ with a typical nacre‐like structure.^[^ ^30^ ^]^

To simultaneously address the shortcomings of MXene and BN as well as PDMS, a surface coordination strategy was proposed. In this work, BN was designed to coordinate with MXene to jointly improve the mechanical properties and thermal conductivity of PDMS. It is worth mentioning that Multifunctional fillers of MXene and BN imitating the chestnut‐structure was constructed. Compared with previous work, the unique chestnut‐like structure constructed not only gives the material double‐layer flame retardancy and electromagnetic shielding protection, but also is expected to build an orderly thermal conduction path, which can simultaneously achieve flame retardancy and thermal conductivity of the composite material. Therefore, this work provides a biomimetic method for manufacturing high‐performance composite materials with excellent flame retardancy and thermal conductivity as well as EMI shielding and has great prospects for applications in electronic devices.

## 2 Experimental Section

### 2.1 Materials

Hydrochloric acid (HCl, 37%) and lithium fluoride (LiF, 99.99%) were purchased from Sinopharm Chemical Reagent Co., Ltd. Ti_3_AlC_2_ (MAX, 400 mesh) was provided by Shanghai Aladdin Biochemical Technology Co., Ltd. DI water (Beijing Aoli general chemical reagent sales co., Ltd.) was used as dispersant.

Both the base agent as well as the Sylgard 184 curing agent for PDMS (65.0%) were provided by Dow Corning Co., Ltd. (Shanghai, China). Analytical‐grade sodium hydroxide (NaOH, AR, 96%) was obtained from Beijing Chemical Works. BN (99.5 wt.%, 0.1–0.4 µm) was modified via a hydroxylation substitution reaction performed in a mechanically stirred 5 m NaOH solution for 12 h at a temperature of 100 °C and at a concentration of 5 mg mL^−1^. Next, the NaOH‐treated BN was cleaned with H_2_O and filtered. Cleaning and filtering were repeated until the suspension had a pH value of 7.0. Subsequently, the obtained particles were dried for 12 h at 70 °C in a Petri dish, cooled to room temperature, and held in a desiccator before further use. Other reagents were chemically pure and used without any further purification.

### 2.2 Fabrication of MXene

**Figure 1:**
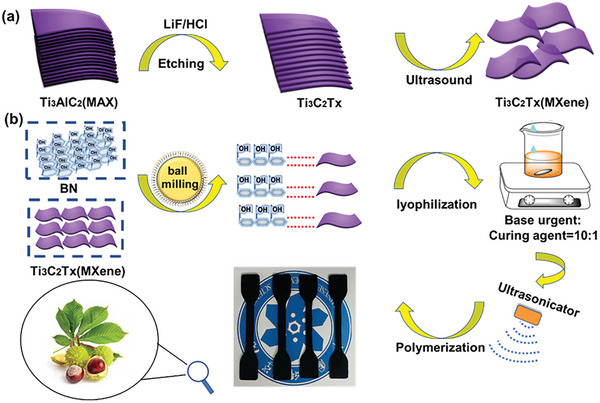
a) Exfoliating process leading to MXene and b) the fabrication of PDMS/MXene@BN composites.

The fabrication processes leading to MXene flexible film were schematically illustrated in **Figure** **1** MXene was synthesized by etching MAX powder in HCl and LiF solutions. Single‐layer/few‐layer samples of MXene were further obtained by ultrasonic treatment. In a typical procedure, 4.8 g LiF was mixed in a Teflon beaker with 60 mL of 12 m HCl for 5 min. Subsequently, 3 g MAX powder was gradually added to this solution and stirred for 48 h at a temperature of 40 °C to perform etching. The slurry that was produced was then cleaned with deionized water and centrifuged. This was repeated until a pH value higher than 6.0 was obtained. The solid product was subsequently sonicated in deionized water for 15 min and then centrifuged for 15 min at 3500 rpm to obtain MXene. To determine the MXene suspension solid content, the solution was evaporated and the remaining solid material was weighed.

### 2.3 Fabrication of MXene@BN

First, MXene and BN were placed into an agate ball mill with 300 mL of a 5:1 mixture of ethanol and deionized water. Milling was performed with a planetary ball mill at 800 rpm for 48 h. Subsequently, the ball mill products were freeze‐dried with a freeze‐dryer to obtain MXene@BN powder.

### 2.4 Fabrication of PDMS/MXene@BN

The synthetic procedure leading to PDMS/MXene@BN is depicted in Figure 1a. The PDMS base and curing agents were mixed together (10:1 ratio of base to curing agent) and stirred for 20 min. Then, ultrasonication was employed to disperse the MXene@BN in the PDMS at different ratios of 0.6, 1.2, and 2.4 wt.%, thus forming a homogeneous mixture. The PDMS/MXene@BN mixture was subsequently placed in a vacuum tank and defoaming was performed until no bubbles produced. Subsequently, PDMS/MXene@BN composite films with various MXene@BN contents of 0.6, 1.2, and 2.4 wt.% were completely dried for 5 h at 60 °C under vacuum. This was followed by cooling to ambient temperature, and the sample with a MXene@BN content of 0.6 wt.% was denoted as PDMS/MXene@BN 0.6. Pure PDMS, PDMS/MXene (with a MXene content of 2.4 wt.%) and PDMS/MXene@BN (1.2 wt.% and 2.4 wt.% MXene@BN) were prepared in a similar manner.

### 2.5 Characterization

The MXene nanosheet surface morphology and thickness were characterized via atomic force microscopy (AFM) using an instrument manufactured by Bruker Dimension Icon (Germany).

The cross‐sectional and surface micromorphology and microstructure of MXene, BN, and PDMS/MXene@BN were studied under a cold field emission scanning electron microscope (SEM, ZEISS Sigma 300, Germany) at 5.0 kV. SEM analysis was coupled with energy‐dispersive X‐ray spectroscopy (EDX).

Crystalline structures were evaluated by X‐ray diffraction (XRD) (Rigaku Smartlab 9 KW, Japan). XRD was performed using Cu Kα radiation with a wavelength of 1.54 Å. The chemical states and composition of each composite film was identified by X‐ray photoelectron spectroscopy (XPS, Thermo Scientific K‐Alpha, United States) using an Al Kα anode.

Functional groups were characterized by Fourier‐transform infrared (FTIR) (Thermo Scientific iN10, United States). Samples were prepared into KBr pellets, spectra were obtained in transmittance mode, and the wavenumber range was 4000–500 cm^−1^.

CMT6103 universal testing machine was used to perform mechanical testing at 10 mm min^−1^. The electrical conductivities of the prepared composite films were evaluated with an RTS‐8 four probe tester.

Thermogravimetric analysis (TGA) was performed under a N_2_ atmosphere from 30–800 °C with a ramp rate of 10 °C min^−1^ (NETZSCH TG 209 F3).

The smoke suppression and combustion behavior of the samples was evaluated with a cone calorimeter test (CCT, GOVMARK) according to ISO 5660 Samples had dimensions of 100 mm ×100 mm × 3.0 mm, the incident flux was 50 kW m^−2^ and the testing distance was 60 mm.

Film thermal conductivities (λ, W m^−1^ K^−1^ were evaluated by performing a guarded heat flow test (DCT 25, TA, USA) following ASTM E1530. Films with diameters of 50 mm were used for this test. Joule heating performance of PDMS/MXene@BN was investigated with a DC power supply (SS‐L3010SPD, A‐BF, China) with a defined voltage, while a thermal imaging camera (Fluke VT08) was used to determine the thermal distribution of the PDMS/MXene@BN heater and measure the surface temperature of the electric heater.

A thermal analyzer (TAQ2000) was used to perform differential scanning calorimetry (DSC) tests under nitrogen. DSC tests were performed at a scan rate of 10 °C min^−1^ in the temperature range of −120 to 200 °C.

Transmission data S21 and reflection coefficient data S11 were obtained with a microwave network analyzer (Agilent PNA‐N5244A). The total EMI‐SE (SET) was calculated with Equation (1), THE multiple internal reflection SE (SEM) was calculated with Equation (2), the absorption SE (SEA) was calculated with Equation (3), and the reflection SE (SER) was calculated with Equation (4):^[^ ^31^ ^]^

(1) $$R={\left|s_{11}\right|}^{2}={\left|s_{22}\right|}^{2},T={\left|s_{12}\right|}^{2}={\left|s_{21}\right|}^{2}$$

(2) A=1−R−T

(3) $${\mathrm{SE}}_{R}=-10\log\left(1-R\right),SE_{A}=-10\log\left(\frac{T}{1-R}\right)$$

(4) $$\;{\mathrm{SE}}_{T}=\;SE_{R}+SE_{A}+SE_{M}$$

The SEM is negligible if SET > 10 dB. To more accurately compare shielding material effectiveness, the effect of the thickness was removed by comparing normalized EMI SE/t values. Then, considering both the density and the thickness of the materials, SSE and SSE/t are calculated by the following:

(5) $$\mathrm{SSE}=\mathrm{EMI}\frac{\mathrm{SE}}{\mathrm{density}}=\mathrm{dB}\;cm^{3}\cdot g^{-1}$$

(6) $$\mathrm{SSE}/t=\mathrm{SSE}/\mathrm{thickness}={\mathrm{dB}\;\mathrm{cm}}^{2}\cdot g^{-1}$$

The ability to block waves is expressed as the EMI shielding efficiency (%), via Equation (7):

(7) $$\mathrm{Shielding}\;\mathrm{efficiency}\;\left(\%\right)=100-\left(\frac{1}{10^{\frac{\mathrm{SE}}{10}}}\right)\times 100\%$$

The electric heater surface temperature and composite film heater thermal distribution were measured with a thermal imaging camera (Fluke VT08).

## 3 Results and Discussion

### 3.1 Characterization of MAX and MXene

**Figure 2:**
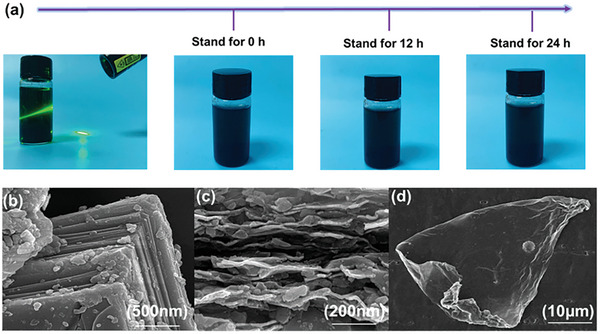
a) MXene nanosheet Tyndall scattering effect and stability of exfoliated MXene suspensions. SEM micrographs of b) MAX, c) multilayered Mxene, and d) MXene.

**Figure 3:**
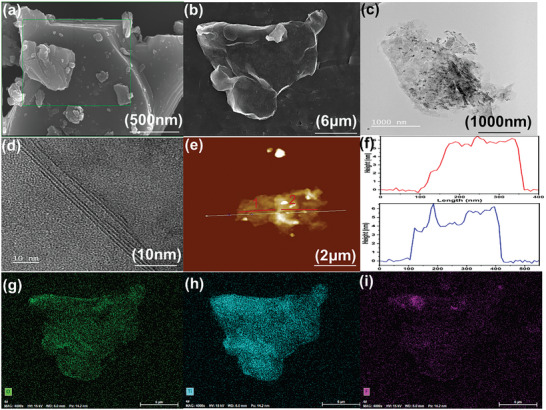
a) SEM image of MAX. b) SEM image of MXene and g–i). distribution diagrams of element in (b). c) TEM, d) HRTEM, And e) AFM images of MXene (Ti_3_C_2_T_x_), f) Corresponding heights of line 1 and line 2 from (e).

The MXene were hydrophilic and dispersible, as is demonstrated by the Tyndall effect in **Figure** **2**a. Owing to of its surface polar groups and the MXene structure, the exfoliated MXene suspension remained stable in the dispersion for as long as 24 h without precipitation, thus further demonstrating that this filler had high dispersibility and stability. Figure 2(b–d) show the schematic illustration of the exfoliated MXene. Figure **3**a, shows that the unmodified Max crystal has a typical multi‐layer structure.^[^ ^32^ ^]^ In contrast, Figure 3b shows the relatively thin “few‐layer” structure of the MXene sample. High‐resolution TEM micrographs of these MXene are provided in Figure 3c,d. It is evident that the prepared few layer MXene are very thin, with only about 2–3 layers of MXene. The AFM data are displayed in Figure 3e,f, indicating that MXene comprised of thin layer nanosheets have relatively smooth surfaces, with an average thickness in the range of ≈4.01 to 5.23 nm. A single‐layer MXene is generally assumed to have a thickness of 1.53 nm.^[^ ^33^ ^]^ Therefore, the exfoliated MXene sample in this experiment has a thickness of about 2–3 layers. This is in good agreement with the HRTEM image displayed Figure 3d and indicates the successful preparation of the few‐layer MXene. Figure 3g–i show the elemental content of oxygen, titanium, and fluorine. The presence of F and O can be detected in randomly selected points, once again indicating the successful preparation of few‐layer MXene. It also clearly shows that all elements completely cover the test area, indicating that the MXene have good dispersibility.

### 3.2 Characterization of the MXene@BN

**Figure 4:**
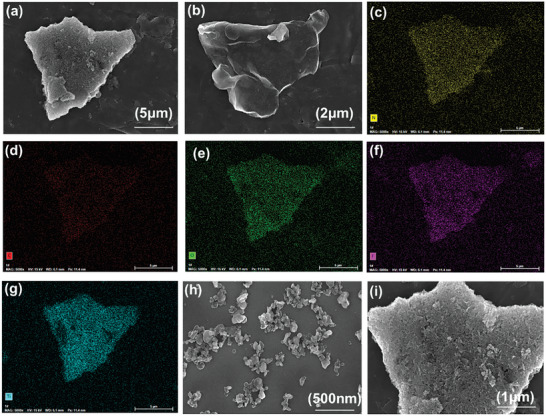
MXene@BN hybrid EDX analysis: a) SEM images of MXene@BN and MXene (Ti_3_C_2_T_x_). Mapping images: c) nitrogen(N), d) carbon (C), e) oxygen (O), f) fluorine (F), and g) titanium (Ti), h) SEM image of BN. i) SEM image of MXene@BN hybrids.

The MXene@BN hybrids were analyzed by SEM, as shown in **Figure** **4**a. After ball milling had been performed to mix the MXene, the BN structure was retained. After ball milling, the majority of the functional BN plates are connected to the wrinkled and thin MXene. It can be clearly seen that MXene wrap a smaller amount of BN. Figure 4i shows there are some white substances around MXene@BN, which has a rougher surface than that of a MXene, indicating that BN had been successfully adsorbed on the edge of MXene. The interaction between the MXene layers is weakened and MXene peeling is promoted by BN adsorption on the MXene surface. Thus, the size of the of MXene@BN particles is relatively small. A disc‐like morphology can be observed in the SEM image of BN (Figure 4h). Figure 4c–g shows the elemental maps of nitrogen, carbon, oxygen, fluorine, and titanium, which clearly reveal that all elements completely cover the test area, thus indicating the successful preparation of MXene@BN hybrids.

**Figure 5:**
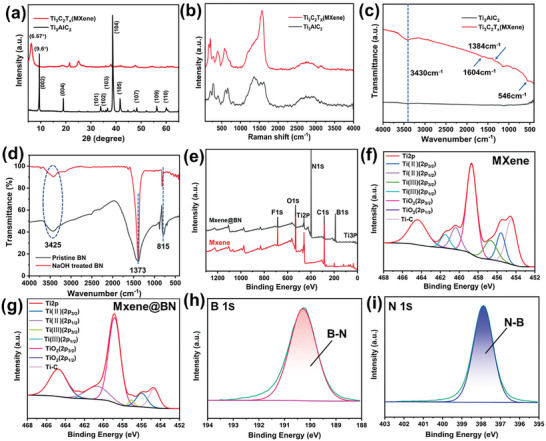
a) XRD patterns, b) Raman spectra, and c) FTIR spectra of MAX (Ti_3_AlC_2_) and MXene (Ti_3_C_2_T_x_). d) FTIR spectra of BN and NaOH‐treated BN. e) XPS survey spectra of MXene and MXene@BN. High‐resolution Ti 2p spectra of f) MXene (Ti_3_C_2_T_x_) and g) MXene@BN. h) B 1s spectrum i) and N 1s spectrum of MXene@BN.

As indicated by the XRD patterns in **Figure** **5**a, the (002) peak significantly shifts from 9.60° to 6.67°, indicating the successful preparation of the few‐layered MXene structure. This peak shift implied that the crystal plane widened from 9.20 Å to 13.06 Å. Compared to MXene, the sharp (104) peak at 2*θ* ≈ 39° disappeared in the XRD pattern of MAX, which was attributed to the removal of aluminum. Meanwhile, the (002) peak corresponding to the MAX phase was shifted to a lower 2*θ* value and was broader. This indicated that the Al layers were etched away, leading to the introduction of polar groups onto the surface such as –F, –O, and –OH. Figure 5b shows Raman shift spectra of MAX and MXene. Both spectra show the same peaks, indicating that the vibrational characteristics are not affected by the exfoliation of MAX. MAX and MXene were also studied by FTIR spectroscopy, as displayed in Figure 5c. The peak at 1384 cm^−1^ indicates the presence of C–F bonds. Bands at 3430, 1604, and 546 cm^−1^ are related to –OH bond vibrations. The FTIR spectra of pristine BN and NaOH‐treated BN are displayed in Figure 5d. Absorption peaks at 815 cm^−1^ and 1373 cm^−1^ respectively correspond to out‐of‐plane B‐N‐B bending vibrations and in‐plane BN stretching vibrations. Meanwhile, the stretching vibrations of hydroxyl groups are indicated by the broad band at a wavenumber of 3425 cm^−1^. Treatment with NaOH significantly strengthens this band, demonstrating successful BN hydroxylation. MXene and the MXene@BN were evaluated by XPS, and their survey spectra are displayed in Figure 5e. These spectra show peaks ascribed to C, Ti, O, and F, indicating the successful preparation of MXene. Meanwhile, it is particularly noteworthy that the spectrum of MXene@BN displays N 1s and B 1s peaks. Figure 5f,g shows the high‐resolution Ti 2p spectra of MXene and MXene@BN. The existence of low‐valence Ti species is indicated by these spectra. The Ti (II) (2p3/2) signal can be observed at ≈455.8 eV, that of Ti (II) (2p1/2) is at ≈460.8 eV, that of Ti (III) (2p3/2) is at ≈456.8 eV, and that of Ti (III) (2p1/2) is located at ≈462.0 eV. Moreover, Figure 5h,i displays the high‐resolution N 1s and B 1s spectra of the MXene@BN hybrid. The binding energy of the N−B bond corresponds to the characteristic peak of BN.^[^ ^34^, ^35^ ^]^ The B 1s spectrum (Figure 5h) in the MXene@BN hybrid can be resolved into one peak at 190.43 eV, which can be ascribed to B−N groups. The N 1s spectrum of MXene@BN (Figure 5i) can be fitted by one peak at 398.75 eV, which can be attributed to the N−B functional groups.

**Figure 6:**
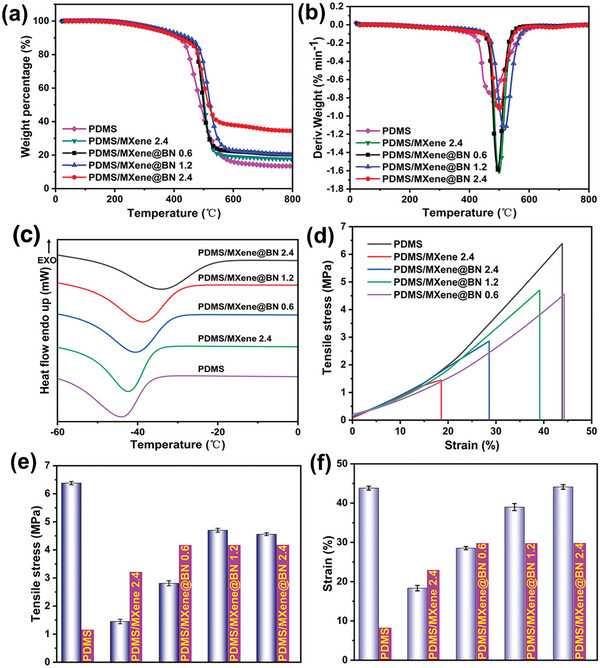
a) TGA, b) DTG, and c) DSC curves of pure PDMS and the modified PDMS/MXene@BN samples. d) Tensile stress–strain curves, e) tensile strengths, and f) strain strengths of PDMS and PDMS/MXene@BN.

**Figure** **6**a show that with increasing MXene@BN loading, the glass transition temperature (*T*_g_) of the PDMS/MXene@BN samples gradually increase. As indicated by the TGA curves in Figure 6b, the thermal degradation trends of PDMS and its composites are very similar, and there is only one step of thermal degradation process, which mainly occurs between 400 and 500 °C. Compared with pure PDMS, the original decomposition temperature and residual carbon content of PDMS composites increased significantly when the MXene or MXene@BN were added. This was because the MXene and MXene@BN are more thermally stable than pure PDMS. With increasing MXene@BN content from 0.6 to 2.4 wt.%, the initial decomposition temperature of PDMS/MXene@BN composites increased from 418.3 for pure PDMS to 453.8, 458.8 and 461.6 °C respectively, and the carbon content also increased to 20.44%, 20.90% and 34.79% respectively. However, for PDMS/MXene 2.4, its initial decomposition temperature was only 439.9 °C, and the residual carbon content was 17.74%, which was only 4.39% higher than the residual carbon content of unmodified PDMS, and its increase was far lower than that observed for PDMS/MXene@BN 2.4. The enhanced thermal resistance of PDMS is mainly attributed to the synergistic contributions of BN and MXene.

The DTG curves of the composite materials are highly consistent with the curve of pure PDMS, as indicated in Figure 6b The maximum degradation rate occurs near 495.4 °C. The only difference is that the composites exhibit relatively low maximum thermal degradation rates, as the high thermal stability of MXene and MXene@BN inhibits thermal degradation.

The DSC curves are shown in Figure 6c With 2.4 wt.% MXene@BN, the PDMS/MXene@BN composite exhibits a *T*_g_ value of −43.4 °C, while the *T*_g_ value of the unmodified PDMS is 5.7 °C lower. With increasing MXene@BN loading, the hard two‐phase interfacial layer between the PDMS polymer matrix and MXene@BN increases. The greater presence of this interfacial layer means that PDMS molecular chain movement is restricted, leading to a higher *T*_g_ value.^[^ ^36^ ^]^

We further studied the effect of PDMS on its mechanical properties. As shown in Figure 6d, for pure PDMS, its tensile strength is 6.38 ± 0.06 MPa. When 2.4 wt.% MXene are directly added to PDMS, their tensile strength decreases to 1.5 ± 0.08 MPa, which is due to the fact that pure MXene readily agglomerate in PDMS and reduce the crosslinking degree of the curing system. Compared with pure PDMS, when the content of MXene@BN in PDMS increases from 0.6 to 2.4 wt.%, its tensile strength decreases by 1.82%, 1.68%, and 3.57%, respectively. This trend is mainly attributed to the relationship between MXene@BN and PDMS matrix that results in two‐phase interfaces (weak interface connections). The internal parts of PDMS composites are prone to microcracks and voids, resulting in a decrease for the tensile strength.

The internal defects in composite materials can become stress concentration points when subjected to an external force. This can lead to the rapid expansion and fracture of internal microcracks, leading to a reduction in tensile strength and elongation at break. However, when the MXene@BN content of the composite samples was reduced from 2.4 to 0.6 wt.%, the tensile strength increased by 1.36 ± 0.4%, 3.25 ± 0.9%, and 3.11 ± 0.6%, respectively. There are two reasons for this trend. One, the shear force generated by the ball milling shear method disrupts the van der Waals force between the MXene layers, thereby improving the compatibility, making the reaction of the PDMS curing system more complete, and significantly improving the crosslinking density. Two, the functionalization of BN on MXene can improve MXene dispersion within the PDMS matrix and limit the adverse effects of MXene on the curing reaction.

### 3.3 Fire Performance

**Figure 7:**
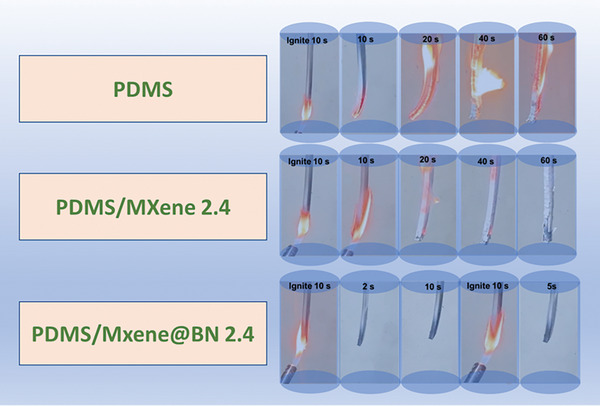
Combustion testing of unmodified PDMS, PDMS/MXene, and PDMS/MXene@BN 2.4 composites.

Combustion testing was performed to provide additional confirmation regarding the role of MXene @BN. From **Figure** **7**, it is evident that after continuous ignition for 10 s, pure PDMS will burn violently and be accompanied by thick black smoke. When the flame burns for 20 s, the sample begins to bend and deform, and severe dripping occurs, indicating that PDMS has high flammability. A similar situation occurred during the combustion of PDMS/MXene. Compared to pure PDMS and PDMS/MXene 2.4, PDMS/MXene@BN 2.4 has excellent flame retardancy. After continuous ignition for 10 s, it will automatically extinguish within 2 s and carry a small amount of white smoke. After igniting it again for 10 s, it can still automatically extinguish within 5 s, with almost no smoke being generated. The comparison data provided further confirmation that the presence of MXene@BN in the PDMS composite provided superior flame retardancy. Notably, the PDMS composite did not readily combust, likely due to the protection offered by the novel bilayer protection which inhibited the spread of the flame.

**Figure 8:**
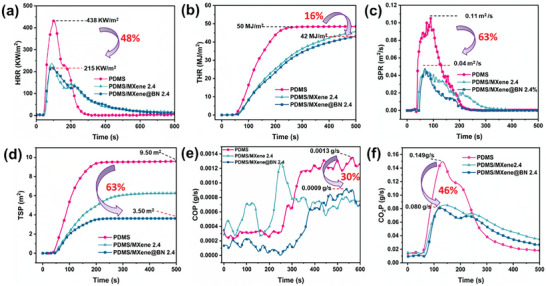
a) Heat release rates (HRR); b) total heat release (THR); c) smoke production rates (SPR); d) total smoke production (TSP); e) CO production (COP); and f) CO_2_ production (CO_2_P) curves of unmodified PDMS, PDMS/MXene 2.4 and PDMS/MXene@BN 2.4 composite samples.

Benefiting from the protection offered by the bilayer structure, the PDMS/MXene@BN composite materials are highly flame retardant. To objectively study their combustion behavior, the cone calorimeter method was utilized to simulate a real fire environment. Unmodified PDMS exhibited a fast ignition time of ≈33 s and achieved a PHHR of 438 kW m^−2^ at 70 s under a heat flow rate of 35 kW/m^2^. The THR of PDMS eventually reached 50 MJ m^−2^ under this heat flow rate (**Figure** **8**a,b). Meanwhile, PDMS/MXene@BN 2.4 required a significantly longer 40 s for ignition. This composite had PHHR and THR values of 215 kW m^−2^ at 90 s and 42 MJ m^−2^, which were 48.03% and 16.51% lower than that of unmodified PDMS. In addition, PDMS/MXene@BN 2.4 showed superb smoke suppression performance, with an SPR of 0.04 m^2^ s^−1^ and TSP of 3.51 m^2^. Compared with the SPR and TSP values of unmodified PDMS, this represented a significant reduction of 63.20% and 63.50%, respectively (Figure 8c,d). The better performance of the PDMS/MXene@BN 2.4 composite was attributed to its BN nanosheets providing a physical barrier effect and MXene offering a shielding effect. Furthermore, PDMS/MXene@BN 2.4 exhibited the lowest COP value (0.0009 g s^−1^) during combustion, indicating the excellent detoxification effect and smoke suppression performance of this composite material (Figure 8e). Thus, the production of toxic CO was suppressed by the adsorption properties and catalytic effect of BN, particularly when the flammability and smoke suppression performance of the PDMS/MXene@BN composite materials are compared to that of PDMS/MXene. **Table** **1** lists some previous results on the filler efficiency to compare our findings with previous studies. The data indicates the filler efficiency in our work is indeed remarkable, which demonstrates a quite high flame‐retardant efficiency can be obtained by introducing a lower content of MXene@BN into PDMS.

**Table 1:** The filler efficiency of previous literature and that of our work.

Materials	Filler Fraction [wt.%]	φPHRR	φTHR	φSPR	φTSP	Reference
PP/MP‐PEPA	39.7	2.01%				[37]
PP/MCU@APP	30.0	1.75%				[38]
CNF/GO/SEP/BA	23.0	1.10%				[39]
PUF/CH/APP/CH/PAA‐KAO	17.8	3.75%	2.23%		3.33%	[40]
EVA/PD‐LDH@MF	15.0	4.60%	1.64%			[41]
Ramie fabrics/APP/PEI	13.8	4.88%	3.27%			[42]
PU/MH‐APP	13.7					[43]
EP/Ionic liquid‐based metal–organic hybrid	6.0	5.16%	0.17%	3.85%	2.56%	[44]
EP/Frs@rGO	5.0	7.01%				[45]
PS/g‐C_3_N_4_/OAHPi hybrids	4.0	6.93%	2.39%	5.47%		[46]
EP/1‐vinyl‐3‐(diethoxyphosphoryl)‐propylimidazolium bromide	4.0	16.25%	3.52%			[47]
WPU/BP/G	3.55	13.56%	10.88%			[48]
EP/Fr@PZS	3.0	15.34%	9.03%			[49]
PUA/BP@EC/Exf	3.0	14.84%	11.50%			[50]
PS/FGO	2.0	18.00%	6.89%			[51]
PDMS/MXene@BN	2.4	20%	6.66%	26.25%	26.25%	This work

**Figure 9:**
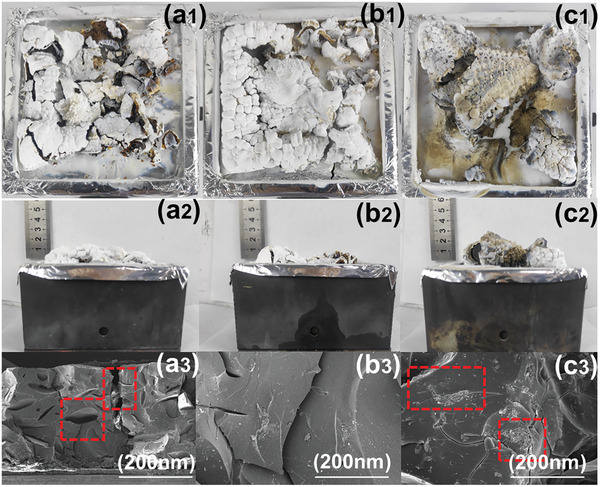
Digital photos showing top view of residual char: a_1_) pure PDMS; (b_1_) the PDMS/MXene 2.4 composites. (c_1_) the PDMS/MXene@BN 2.4 composites; Side view of the outer char layer: a_2_) pure PDMS; b_2_) the PDMS/MXene 2.4 composites; c_2_) the PDMS/MXene@BN 2.4 composites. SEM images of residual char: a_3_) pure PDMS; b_3_) the PDMS/MXene 2.4 composites; and c_3_) the PDMS/MXene@BN 2.4 composites.

For pure PDMS, the carbon slag surface is very loose and has some large pores (**Figure** **9** (a_1_‐a_2_‐a_3_)). In contrast, the carbon layer of the PDMS/MXene 2.4 composite material exhibits a relatively dense structure with only a small number of small pores (Figure 9 (b_1_‐b_2_‐b_3_)). The pores on the surface of the residual carbon in PDMS/MXene@BN 2.4 are significantly reduced and exhibit denser and more compact structure (Figure 9 (c_1_‐c_2_‐c_3_)). The formation of the carbon layer can impede the transfer of heat and the release of combustibles, thereby achieving the goal of fire prevention.

### 3.4 Flame Retardancy Mechanism

**Figure 10:**
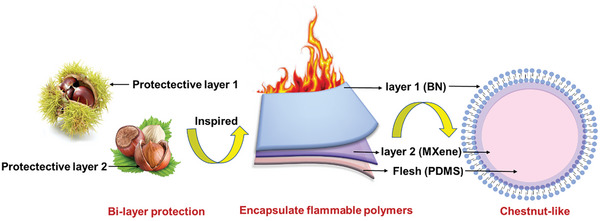
Schematic diagram showing the chestnut‐like structure of PDMS/MXene@ BN and its flame retardancy mechanism.

Through the above research and analysis, Figure **10** proposes a prediction of the flame retardancy and smoke suppression mechanism of PDMS/MXene@BN composite material. Due to its chestnut‐like morphology, this composite material is wrapped by two layers encapsulate and protect the flammable PDMS from external flames. MXene act as a thermally conductive layer and barrier.^[^ ^52^ ^]^ The presence of these nanosheets leads to the rapid transfer of heat, meaning that high local temperatures are avoided. They also block oxygen molecules.^[^ ^53^ ^]^ Moreover, the hydroxylation pretreatment of BN enhances BN nanosheet adsorption on the MXene surface. Its flame retardancy mechanism mainly involves the physical thermal barrier effect, as it forms an insulating layer that acts as a barrier to combustible gases and heat transfer. The physical barrier effect of BN nanosheets retards the transfer of both mass and heat between the flame zone and PDMS substrate, as well as the escape of pyrolytic volatiles, thereby reducing smoke toxicity and heat release.^[^ ^54^ ^]^ Furthermore, the spacing between the MXene was also significantly increased. This is because the BN was distributed on the surface of the MXene, which acted as a barrier and effectively prevented MXene from back‐stacking.^[^ ^55^ ^]^

### 3.5 EMI Shielding Performance

**Figure 11:**
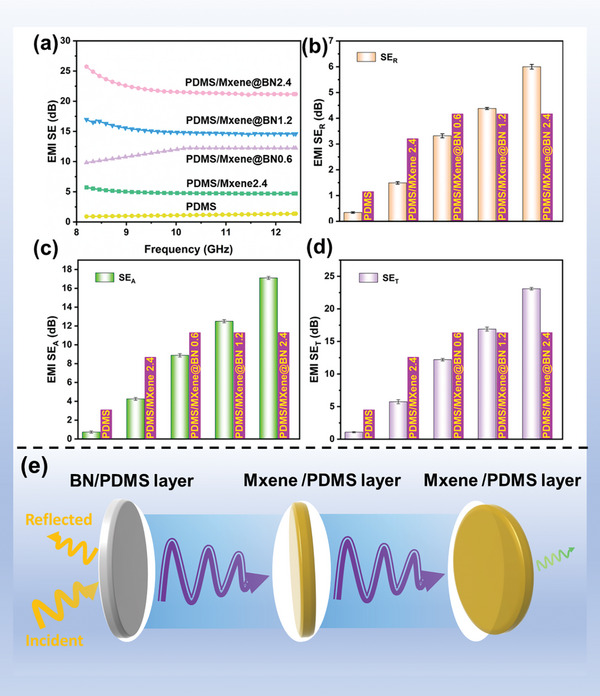
a) EMI SE values of different multilayer films in the frequency range of 8.0–12.5 GHz. b–d) SE_R_, SE_A_ and SE_T_ values of different multilayer films at 8.3 GHz, respectively. e) EMI shielding mechanism of the prepared bilayer films.

The EMI shielding behavior of the PDMS /MXene@BN composite film was studied by measuring its EMI SE in the frequency range of 8.0–12.5 GHz as shown in **Figure** **11**a. Throughout the X‐band, the EMI SE of the PDMS/ MXene@BN composite film slightly varies with changing frequency and increases with increasing MXene@BN loading. The electrical conductivity and EMI shielding performance of materials are inversely proportional. The unmodified PDMS films exhibit excellent electrical insulating capabilities and are transparent with regard to EMI radiation.  The agglomeration effect of MXene after ball milling is not significant, and thus it plays a superior electromagnetic shielding role in PDMS. With a maximum EMI SE of 26.3 dB at 8.5 GHz, the PDMS/MXene@BN 2.4 film exhibits superb performance. In order to better compare our work with previous studies, Table S1 (Supporting Information) summarizes previous reports on the efficiency of various fillers for electromagnetic shielding properties. The data show that the SE are indeed very high in our PDMS/ MXene@BN composites.

To explore their EMI shielding mechanism, the absorption SE (SE_A_), reflected SE (SE_R_), and total EMI SE (SE_T_) values of the multilayer films were determined, as shown in Figure 11a–d. The SE_A_ values of the multilayer films are significantly higher than their SE_R_ values. With increasing MXene@BN content, the SE_R_ exhibited a smaller change, and SE_A_ significantly increased. This behavior suggests that that the primary mechanism through which the EMI shielding is achieved is via microwave absorption. Figure 11e shows a schematic depiction of the EMI shielding mechanism of these multilayer film materials. Initially, BN/PDMS is directly penetrated by incident electromagnetic waves. When they encounter MXene/PDMS, some of the electromagnetic radiation is immediately deflected by the highly conductive surface of the MXene.^[^ ^56^ ^]^ The remaining electromagnetic waves enter the PDMS interior.^[^ ^57^ ^]^ Electromagnetic waves are both reflected and absorbed by the conductive MXene/PDMS material, attenuating the remaining waves.^[^ ^58^ ^]^ Consequently, the MXene@BN loading in the PDMS composite influences the EMI SE.

### 3.6 Thermal Conductivity

**Figure 12:**
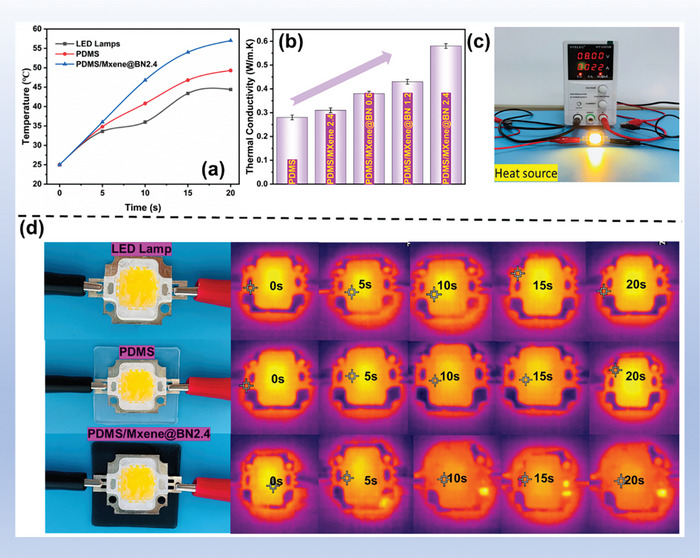
a) Surface temperature variation curves of an LED lamp, LED lamp with PDMS film, and LED lamp with PDMS/MXene@BN 2.4 film. b) Thermal conductivity of the LED lamp; c) Digital photograph of the experimental device used to evaluate thermal conductivity. d) IR images showing changes in the surface temperature over time of the bare LED lamp and LED lamp with the PDMS or PDMS/MXene@BN film.

The temperatures of the PDMS and PDMS/MXene@BN films, were recorded with an infrared camera using a LED light as a heat source to study their heat transfer capacities.  The same LED light was used for both films, and measurements were performed under a constant voltage and current (8.0 V, 0.022 A). The experimental results are shown in **Figure** **12** clearly, the characteristics of the filler significantly influence the thermal conductivity of these films. The polymer chains were highly disordered and consequently the phonons were scattered in the polymer matrix.^[^ ^59^ ^]^ Thus, the unmodified PDMS film had a low thermal conductivity of 0.21 W m^−1^K^−1^ (Figure 12 (b)). With a MXene@BN loading of 2.4 wt.%, the thermal conductivity of BN@MXene/PDMS 2.4 is 0.59 W m^−1^K^−1^, which is 210% higher than the thermal conductivity of the PDMS film. This is because continuous thermal conduction channels are provided by the honeycomb‐like morphology of the composite, allowing for the more rapid transfer of heat compared to an amorphous filler. Furthermore, compared with the reported thermal conductive composite material (Table S2, Supporting Information), the thermal conductivity of the material we made is significantly.

Surface temperature curves and infrared thermal images of the experimental device are presented in Figure 12(a,c,d). The surface temperature of the PDMS/MXene@BN film increases more quickly than that of the PDMS film.  For PDMS film and PDMS/MXene@BN film in Figure 12a, the surface temperature rises to 47.5 °C and 58.0 °C respectively in the 20 s after the LED light was turned on, while the surface temperature of LED rise to 43.2 °C in 20 s. Hence, these findings reveal that the PDMS/MXene@BN films dramatically outperform the PDMS films in terms of their heat capacity.

### 3.7 Thermally Conductive Mechanism

**Figure 13:**
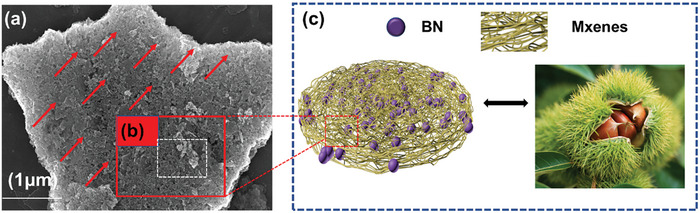
a,b) Thermal conductivity mechanism of PDMS/MXene@BN composites. c) Diagram showing the structure of the PDMS/MXene@BN composite, which resembles that of a chestnut.

As shown in the surface SEM images of **Figure** **13**a, MXene and BN are combined through hydrogen bonding, with MXene@BN arranging vertically along the PDMS composite material, so that an outer protective structure similar to a “chestnut shell” can be successfully obtained. The layered oriented structure shown in Figure 13c can serve as an effective heat transfer channel in the vertical direction of phonons. In addition, compared to other composite material heat conduction path designs (such as 3D network structures prepared by the ice template method,^[^ ^60^ ^]^ threshold network structures,^[^ ^61^ ^]^ etc.), the key characteristic of this study is the use of ball milling technology to combine BN and MXene, which maximizes the construction of an ultra‐efficient thermal conductivity network within inside the composite material at the filler/filler and filler/polymer interfaces.^[^ ^62^ ^]^

## 4 Conclusion

In summary, bioinspired bilayer architectures were synthesized by surface interaction between BN and MXene. Multifunctional PDMS/MXene@BN nanocomposite films with a chestnut‐like shell were successfully prepared via a facile ball milling method, and the integrant was constituted by BN nanoparticles and few layer MXene nanosheets. The incorporation of BN nanoparticles was homogeneously distributed to the MXene nanosheets, which led to formation of a stabilized chestnut shell skeleton architecture of the PDMS film through surface interaction. The PDMS/MXene@BN 2.4 composite was highly thermally conductive (0.59 Wm^−1^K^−1^) and this composite also exhibited improved flame retardancy. Interestingly, the attractive flame retardancy is caused by the MXene and BN protective layers. Additionally, the PDMS/MXene@BN 2.4 film exhibits superb X‐band EMI SE, with the highest value of 26.3 dB achieved at 8.5 GHz. This research offers a new direction for the combination of MXene with BN nanoparticles as a multi‐functional filler for polymers.

## Conflict of Interest

The authors declare no conflict of interest.

## Supporting information

Supporting Information

## Data Availability

The data that support the findings of this study are available from the corresponding author upon reasonable request.
