# Supplementary material for: Facile Construction of Chestnut‐Like Structural Fireproof PDMS/Mxene@BN for Advanced Thermal Management and Electromagnetic Shielding Applications
Source: Adv Sci (Weinh). 2024 Feb 11;11(15):2307482. doi: 10.1002/advs.202307482 (PMC11022730; doi:10.1002/advs.202307482)
Supplement: Supplementary file 1 — Supporting Information [file ADVS-11-2307482-s001.pdf]

ADVANCED SCIENCE
Open Access

Supporting Information

for *Adv. Sci.*, DOI 10.1002/advs.202307482

Facile Construction of Chestnut-Like Structural Fireproof PDMS/Mxene@BN for Advanced Thermal Management and Electromagnetic Shielding Applications

*Fuping Bian, Rui Huang, Xiaobin Li, Jiwen Hu and Shudong Lin**

Supporting information

Facile Construction of Chestnut-like Structural Fireproof PDMS/MXene@BN for Advanced Thermal Management and Electromagnetic Shielding Applications

Fuping Bian[a,b], Rui Huang [a,b], Xiaobin Li[a,b], Jiwen Hu[a,b,c,d,e], Shudong Lin[a,b,c,d,e,*]

[a] Guangzhou Institute of Chemistry, Chinese Academy of Sciences, Guangzhou 510650, P. R. China

[b] University of Chinese Academy of Sciences, Beijing 100049, P. R. China

[c] CAS Engineering Laboratory for Special Fine Chemicals, Guangzhou 510650, P. R. China

[d] CASH GCC Shaoguan Research Institute of Advanced Materials, Nanxiong 512400, P. R. China

[e] CASH GCC Fine Chemicals Incubator (Nanxiong) Co., Ltd, Nanxiong 512400, P. R. China

* Corresponding authors: Guangzhou Institute of Chemistry, Chinese Academy of Sciences, Guangzhou 510650, P. R. China

E-mail addresses: linsd@gic.ac.cn (Shudong Lin)

3.1. Characterization of MAX and MXene

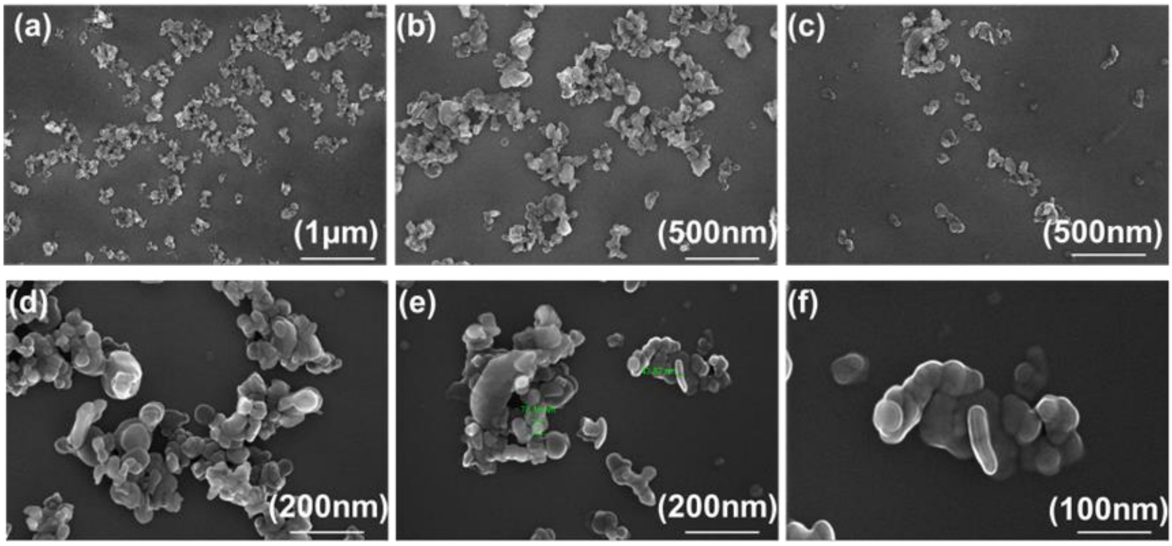

Fig. S1. SEM image of BN-treated NaOH.

3. Results and discussion

Table S1. Performance comparison with other electromagnetic shielding materials.

Filler	Matrix	Content (wt.%)	SE (dB)	Reference
BaM	GNP	10	17.2	[1]
CNT	PP	5	15-20	[2]
CNT	PP	5.6	13	[3]
NCGF	PP	12	23	[4]
CF	PP	18	19.8	[5]
CF+CNT	PP	31	16	[6]
Graphene	PDMS	0.8	20	[7]
Graphene	PEI	10	13	[8]
Graphene@Fe_3O_4	PEI	10	17	[9]
Graphene	PMMA	1.8	19	[10]
Nickel particles	PES	9.4	23	[11]
CNF	PS	15	19	[12]
**MXene@BN**	**PDMS**	**2.4**	**26.3**	**This work**

Table S2. The contrast of thermal conductivity for composites reported in literatures.

Filler	Content (wt.%)	Matrix	Thermal conductivity (W/ (m·K))	Reference
MXene	1	EP	0.587	[13]
MXene	2	TPU	0.148	[14]
MXene	5	PVDF	0.363	[15]
Al_2O_3	10	PS	0.18	[16]
AlN	10	PTFE	0.30	[17]
SiO_2	10	Silicone rubber	0.309	[18]
ZnO-NP	44.3	PI	0.36	[19]
Ni	6	PVDF	0.32	[20]
Si_3N_4	5	Polyethylene	0.25	[21]
SWNT	7.3	PMMA	0.35	[22]
GON	5.0	Liquid paraffin	0.3	[23]
BNNSs	15	Silicone rubber	0.16	[24]
GNP	5	Cyclic butylene terephthalate	0.35	[25]
Graphite	12	Polypropylene	0.34	[26]
**MXene@BN**	**2.4**	**PDMS**	**0.59**	**This work**

References

[1] R. K. Bheema, A. K. Ojha, A. V. Praveen Kumar, K. C. Etika, *J. Mater. Sci.* 2022, *57* (19), 8714.

[2] Ş. Ursache, R. C. Ciobanu, V. Scarlatache, A. Niagu, *Advanced Engineering Forum* 2013, *8-9*, 353.

[3] Y. Li, U. Sundararaj, *AlChE J.* 2014, *61* (1), 296.

[4] Y. Xu, Y. Yang, D. Yan, H. Duan, C. Dong, G. Zhao, Y. Liu, *J. Mater. Sci.: Mater. Electron.* 2017, *28* (12), 9126.

[5] A. Ameli, P. U. Jung, C. B. Park, *Carbon* 2013, *60*, 379.

[6] M.-S. Hong, W.-K. Choi, K.-H. An, S.-J. Kang, S.-J. Park, Y. S. Lee, B.-J. Kim, *J. Ind. Eng. Chem.* 2014, *20* (5), 3901.

[7] Z. Chen, C. Xu, C. Ma, W. Ren, H.-M. Cheng, *Adv. Mater.* 2013, *25* (9), 1296.

[8] J. Ling, W. Zhai, W. Feng, B. Shen, J. Zhang, W. Zheng, *ACS Appl. Mater. Interfaces* 2013, *5* (7), 2677.

[9] B. Shen, W. Zhai, M. Tao, J. Ling, W. Zheng, *ACS Appl. Mater. Interfaces* 2013, *5* (21), 11383.

[10] H. B. Zhang, Q. Yan, W. G. Zheng, Z. He, Z. Z. Yu, *ACS Appl. Mater. Interfaces* 2011, *3* (3), 91.

[11] L. Li, D. D. L. Chung, *Polym. Compos.* 2004, *14* (6), 467.

[12] Y. Yang, M. C. Gupta, K. L. Dudley, R. W. Lawrence, *Adv. Mater.* 2005, *17* (16), 1999.

[13] R. Kang, Z. Zhang, L. Guo, J. Cui, Y. Chen, X. Hou, B. Wang, C.-T. Lin, N. Jiang, J. Yu, *Sci. Rep.* **2019**, *9* (1), 9135.

[14] X. Wang, L. Wang, Y. He, M. Wu, A. Zhou, *Polym. Compos*. **2019**, *41* (1), 350.

[15] Y. Cao, Q. Deng, Z. Liu, D. Shen, T. Wang, Q. Huang, S. Du, N. Jiang, C.-T. Lin, J. Yu, *RSC Adv*. **2017**, *7* (33), 20494.

[16] H. Dong, L. Fan, C. Wong, *IEEE*, **2005**, *2*, 1451.

[17] C. Pan, K. Kou, Q. Jia, Y. Zhang, Y. Wang, G. Wu, A. Feng, J. *Mater. Sci*. **2016**, *27*, 11909.

[18] L. Meyer, S. Jayaram, E. A. Cherney, *Dielectr. Electr. Insul.* **2004**, *11*, 620.

[19] D. Yorifuji, S. Ando, *J. Mater. Chem.* **2011**, *21*, 4402.

[20] W. Zhou, Y. Gong, L. Tu, L. Xu, W. Zhao, J. Cai, Y. Zhang, A. Zhou, *J. Alloys Compd.* **2017**, *693*, 1.

[21] W. Zhou, C. Wang, T. Ai, K. Wu, F. Zhao, H. Gu, *Compos. Part A.* **2009**, *40,* 830.

[22] P. Bonnet, D. Sireude, B. Garnier, O. Chauvet, *Appl. Phys. Lett.* **2007**, *91*, 201910.

[23] W. Yu, H. Xie, W. Chen, *J. Appl. Phys.* **2010**, *107*, 094317.

[24] Z. Kuang, Y. Chen, Y. Lu, L. Liu, S. Hu, S. Wen, Y. Mao, L. Zhang, *Small.* **2015**, *11*, 1655.

[25] Y. J. Noh, H. S. Kim, B.-C. Ku, M.-S. Khil, S. Y. Kim, *Adv. Eng. Mater.* **2016**, *18*, 1127.

[26] V. Causin, C. Marega, A. Marigo, G. Ferrara, A. Ferraro, *Eur. Polym. J.* **2006**, *42*, 3153.
